# Outbreak of Chikungunya in the French Caribbean Islands of Martinique and Guadeloupe: Findings from a Hospital-Based Surveillance System (2013–2015)

**DOI:** 10.4269/ajtmh.16-0719

**Published:** 2018-04-23

**Authors:** Frédérique Dorléans, Bruno Hoen, Fatiha Najioullah, Cécile Herrmann-Storck, Kinda Maria Schepers, Sylvie Abel, Isabelle Lamaury, Laurence Fagour, Raymond Césaire, Stéphanie Guyomard, Ruth Troudard, Yvette Adélaïde, Marie-José Romagne, Magguy Davidas, Séverine Rochais, Sylvie Boa, Frédérique de Saint-Alary, Annabel Preira, Patrick Saint-Martin, Amandine Vaidie, Mathilde Melin, Elise Daudens-Vaysse, Jacques Rosine, Alain Blateau, Luisiane Carvalho, Alexandra Septfons, Marie-Claire Paty, Ghislain Leduc, Sylvie Cassadou, Martine Ledrans, André Cabié

**Affiliations:** 1 Santé Publique France, French National Public Health Agency, Regional Unit (Cire), Antilles-Guyane, Saint-Maurice, France;; 2 Infectious Diseases Department, University Hospital, Fort-de-France, Martinique;; 3 Université des Antilles, Faculté de Médecine Hyacinthe Bastaraud, Pointe-à-Pitre, France;; 4 Virology Laboratory, University Hospital, Fort-de-France, Martinique;; 5 Virology Laboratory, University Hospital, Pointe-à-Pitre, Guadeloupe;; 6 Institut Pasteur de Guadeloupe, Les Abymes, Guadeloupe;; 7 Regional Health Authority of Martinique, Fort-de-France, Martinique;; 8 Regional Health Authority of Guadeloupe, Gourbeyre, Guadeloupe;; 9 Santé Publique France, French National Public Health Agency, Saint-Maurice, France;; 10 Infectious Diseases Department, University Hospital, Pointe-à-Pitre, Guadeloupe;; 11 Université des Antilles, Faculté de Médecine Hyacinthe Bastaraud, Fort-de-France, France;; 12 Centre Hospitalier Universitaire de Martinique, INSERM CIC1424, Service de Maladies Infectieuses et Tropicales, Médecine Polyvalente, Fort-de-France, France;; 13 Centre Hospitalier Universitaire de Pointe-à-Pitre, Inserm CIC1424, Service de Maladies Infectieuses et Tropicales, Dermatologie, Médecine Interne, Pointe-à-Pitre, France

## Abstract

Chikungunya virus (CHIKV) emerged in the Caribbean island of Saint-Martin in December 2013. We implemented a hospital-based surveillance system to detect and describe CHIKV cases including severe forms of the infection and deaths in the islands of Martinique and Guadeloupe. A case was defined as a patient with a CHIKV laboratory confirmation cared for in a public hospital for chikungunya for at least 24 hours, and a severe CHIKV case was defined as a CHIKV case presenting one or more organ failures. Sociodemographic, clinical, and laboratory data were collected and cases classified into severe or nonsevere based on medical records. From December 2013 to January 2015, a total of 1,836 hospitalized cases were identified. Rate of hospital admissions for CHIKV infection was 60 per 10,000 suspected clinical CHIKV cases and severity accounted for 12 per 10,000. A total of 74 deaths related to CHIKV infection occurred. Infants and elderly people were more frequently hospitalized compared with others and severity was more frequently reported in elderly subjects and subjects with underlying health condition. Fifteen neonatal infections consecutive to mother-to-child transmission were diagnosed, seven of which were severe. The most vulnerable groups of the population, such as the elderly, infants, individuals with comorbidities, and pregnant women, should remain the main targets of public health priorities.

## INTRODUCTION

Chikungunya fever is a vector-borne viral disease transmitted to humans by infected *Aedes* mosquitoes. Chikungunya virus (CHIKV) is a member of the Alphavirus genus of the *Togaviridae* family. From a preliminary historical hypothesis on suspicion of CHIKV cases described in the Caribbean in 1827–1828 through the first isolation of the virus in 1952–1953 in Tanzania until evidence of recent circulation in the Caribbean, knowledge on the natural history of the disease increased substantially. Simultaneously, enhanced surveillance systems were implemented across countries to detect and manage new health threats.^1,2^ On December 3, 2013, two CHIKV laboratory-confirmed cases were reported in subjects living on the island of Saint-Martin.^3^ They were the first autochthonous CHIKV cases ever recorded in the Americas. Early microbiological investigations demonstrated that the circulating virus isolated in December 2013 belonged to the Asian genotype distinct from the ECSA genotype isolated during the 2005–2006 epidemics in the Indian Ocean.^4^ On December 9, 2013, the World Health Organization launched an epidemiological alert urging Caribbean countries and territories to enhance their surveillance system to improve their capacity of early detection of CHIKV cases.^5^ Because of intense travelers’ traffic within the area, every Caribbean country or territory was considered to be at high risk for CHIKV emergence.^6^ Countries implemented preparedness and response plans to monitor and prevent the risk of CHIKV dissemination through public health measures to fight against *Aedes* mosquitoes.^7–11^

On December 18 and 25, 2013, health authorities reported the first confirmed CHIKV cases in Martinique and Guadeloupe, respectively.^12^ Soon after, neighboring countries started to notify that they were detecting suspected and confirmed CHIKV cases.^13,14^ Previous CHIKV outbreak reports highlighted the substantial impact of CHIKV introduction in naive immune populations. Although the course of the disease is usually benign, atypical, and severe cases, including cases with fatal outcomes, have been described.^15^ From December 2013 onward, the inhabitants of Martinique and Guadeloupe were affected by major CHIKV epidemics. After the end of the epidemics in November 2014 (Guadeloupe) and January 2015 (Martinique), an overall estimated 154,000 suspected clinical CHIKV cases had consulted general practitioners between December 2013 and January 2015, and we estimated that twice as many cases had not sought medical care. It has been estimated that around 308,000 suspected CHIKV cases arose, accounting for approximately 40% of the population of both islands.^16^

In the beginning of the outbreak, little was known about the virulence of the new CHIKV in a setting where the population had no immunity, and no specific treatment was available. Therefore, it was decided to implement a hospital-based surveillance system in both territories. The objectives were to estimate the incidence of hospitalized cases, to assess the burden of severe cases, and to describe features of hospitalized cases. Herein, we focus on the epidemiological and clinical description of hospitalized cases. We assessed the burden of severe cases and deaths related to CHIKV infection in patients admitted to public hospitals throughout the outbreak.

## MATERIALS AND METHODS

### Settings.

Martinique Island and Guadeloupe archipelago belong to the Lesser Antilles in the eastern Caribbean Sea. Their populations are 381,326 and 403,750, respectively (estimations as of January 1, 2014).

### Study type and period.

An active hospital-based surveillance system was implemented in acute and emergency wards of public hospitals in Martinique (Centre Hospitalier Universitaire de Martinique and Centre Hospitalier du Marin) and Guadeloupe (Centre Hospitalier Universitaire de Pointe-à-Pitre and Centre Hospitalier de Basse-Terre) from December 2013 to January 2015. Clinicians, medical microbiologists, public health nurses, and epidemiologists collaborated during the epidemics to identify and document patients admitted to hospital for a CHIKV infection.

### Case definitions.

A *case* was defined as any patient cared for in the emergency room or acute ward of a public hospital in Martinique or Guadeloupe for at least 24 hours for acute CHIKV infection confirmed either by serological testing (Immunoglobulin M antibody titers) or by detecting the presence of CHIKV genome using a real-time reverse transcription polymerase chain reaction (RT-PCR) assays on blood or cerebrospinal fluid samples. Patients admitted to hospital more than 3 weeks after symptoms onset or for a reason not related to CHIKV infection were excluded from this study.

**Figure 1.**
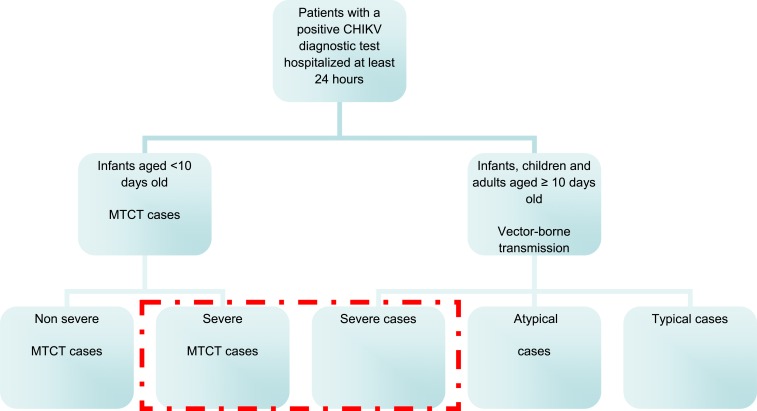
Diagram of classification of clinical forms of chikungunya virus (CHIKV) infected hospitalized patients, Martinique and Guadeloupe - December 2013 to January 2015. This figure appears in color at www.ajtmh.org.

Neonatal CHIKV infections can be vector-borne or result from mother-to-child transmission (MTCT). A MTCT case was defined as a case developing within 10 days of life in a neonate whose mother had confirmed CHIKV infection (RT-PCR) around the time of delivery. We further sorted MTCT cases into two categories: a severe MTCT case had at least one organ failure (respiratory, cardiovascular, cerebral, hepatic, renal, and/or any other organ failure) while a nonsevere MTCT case had no organ failure. Vector-borne cases were assigned to one of the three following categories. A typical case was defined as a case aged 10 days or more and presenting with at least one of the following clinical manifestations: fever, stomach pain, diarrhea, rash, edema, arthralgia, headache, myalgia, itching, or tenosynovitis. An atypical case had at least one additional symptom among the following: severe pain (administration of class III analgesics); neurological manifestations (encephalitis, seizure, or any other neurological disorder); acute cardiovascular disorder (with or without pre-existing cardiac condition); dermatological features (other than rash and/or itching); respiratory, hepatic (hepatic cytolysis), renal or digestive (other than stomach pain and/or diarrhea) disorders; decompensation of a pre-existing comorbidity; and/or hemorrhagic manifestations. A severe case was defined as a *case* developing one or several organ failures in accordance with international consensus definitions: cardiovascular, cerebral, respiratory, renal, hepatic failure, or any other systemic or organ failure (Figure 1).^17^

### Data collection and classification.

A standardized questionnaire was used to collect demographic characteristics, clinical information, and laboratory data. Eligible patients were identified prospectively based on clinical microbiological results in each hospital and further included according to case definitions criteria. Preliminary data were first recorded using a secured on-line sanitary information system (Système d’Information des Maladies Infectieuses Prioritaires). In a second step, public health nurses reviewed medical records of all selected patients and completed a questionnaire for each case. Finally, each case was assigned one of the previously described category and deaths were further classified into those directly or indirectly attributable to CHIKV infection by consensus of infectious diseases physicians.

### Data analysis.

Data analysis was performed using Stata statistical software, version 14.1 (Stata Corp LP, College Station, TX). Incidence of severe cases, mortality, and attack rates were calculated according to populations census data (Martinique, 2013 and Guadeloupe, 2014) and according to estimates of CHIKV clinical infections through sentinel surveillance. Age specific data are available for population census data but are not available for estimated CHIKV clinical infections in the general population. Categorical variables were given as proportions, and continuous variables were described by their mean and range. Typical and atypical vector-borne cases were pooled into nonsevere cases and compared with vector-borne severe cases. Comparisons between categorical variables were performed using tests of proportions and the corresponding *P* values, and comparisons between incidence rates were performed by estimating incidence rate ratios and the corresponding *P* value.

## RESULTS

Overall, we collected 2,024 questionnaires corresponding to as many eligible patients, 1,836 of whom fulfilled the case definitions. Reasons for excluding the 188 remaining patients (9%) were length of hospital stay less than 24 hours (*N* = 26), admission to hospital more than 3 weeks after the onset of symptoms (*N* = 41) or admission for a reason not related to CHIKV infection (*N* = 74), incomplete medical records (*N* = 36), and miscellaneous (*N* = 11).

**Figure 2.**
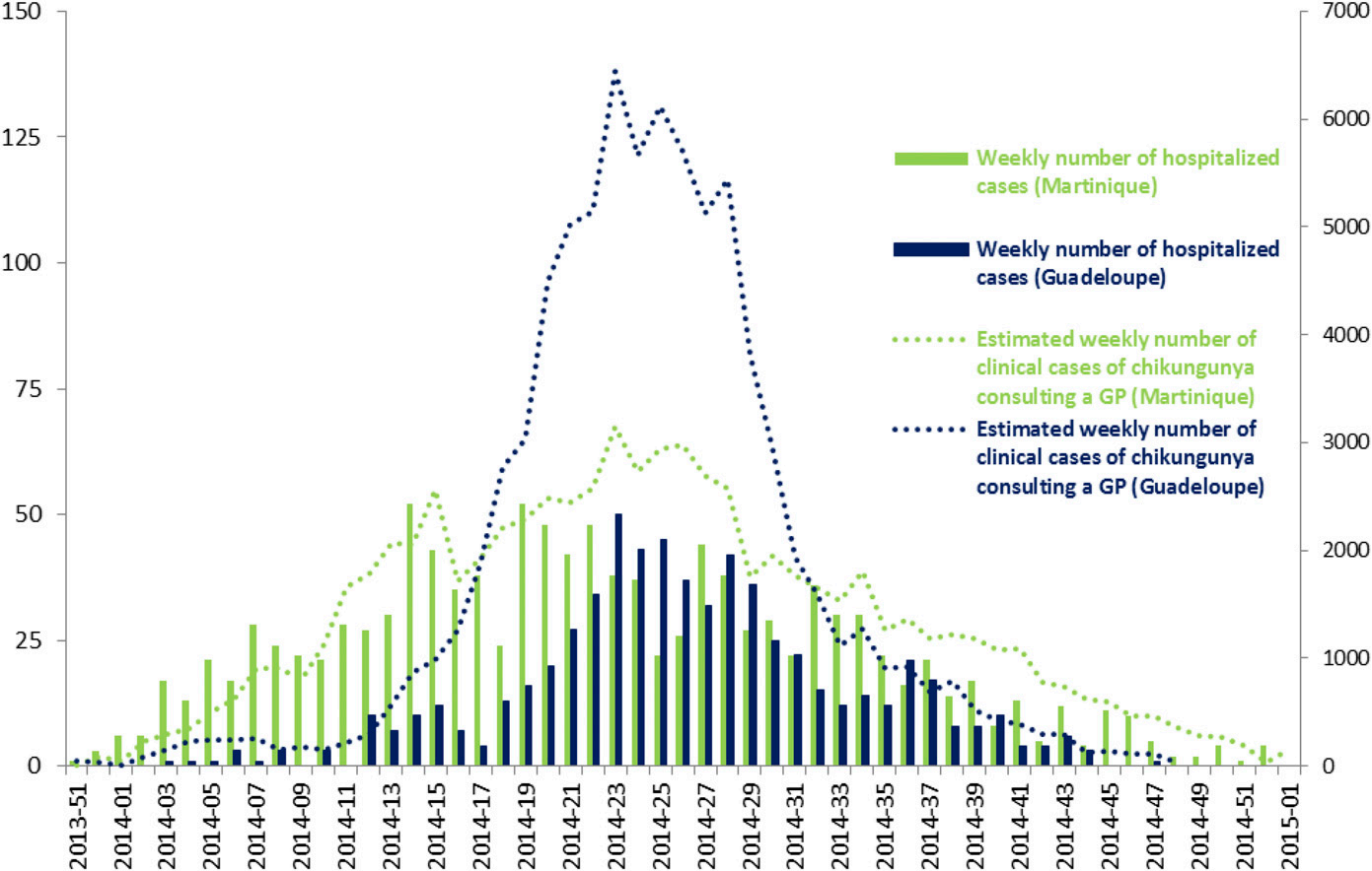
Weekly number of hospitalized chikungunya virus (CHIKV) cases (left axis) and weekly estimated number of clinical CHIKV cases consulting general practitioners (right axis) in the population during the CHIKV epidemics in Martinique and Guadeloupe - December 2013 to January 2015. This figure appears in color at www.ajtmh.org.

Between December 2013 and January 2015, we investigated an overall 1,836 hospitalized cases of which 1,196 cases were in Martinique, and 640 were in Guadeloupe. The number of hospitalized cases peaked in early May 2014 and early June 2014, respectively, in Martinique and Guadeloupe. Hospitalized case curves mirrored that of clinical CHIKV cases occurring in the general population (Figure 2).

### Incidence rates of hospitalization among the total population and among the clinical CHIKV cases.

Among the total population, the overall incidence rate of hospitalization was 23.4 per 10,000 inhabitants. If we estimate our hospitalization rate on the total estimated clinical CHIKV cases that occurred during the epidemic, the attack rate of hospitalization was 60 per 10,000 CHIKV clinical cases.

Of 1,836 hospitalized cases, 1,189 (64.8%) were adults (≥ 15 years old), mean age was 41 years (median: 39 years of age), and sex ratio was 0.9.

### Age-specific incidence rate of hospitalized cases.

We estimated age-stratified incidence rates among age-stratified total population. The highest incidence rates were reported in infants (< 1 year old) and in the elderly (≥ 75 years old) with respective overall rates of 296 per 10,000 and 80 per 10,000 inhabitants. We observed high hospitalization rates in the youngest populations (< 1 year old) in both islands, whereas elderly patients aged 75 years or older were more frequently hospitalized in Martinique (incidence rate: 121 per 10,000 inhabitants) compared with Guadeloupe (incidence rate: 33 per 10,000 inhabitants) (*P* < 0.001) (Table 1).

**Table 1:** Age-specific incidence rates in hospitalized chikungunya virus cases in Martinique and Guadeloupe - December 2013 to January 2015

Age groups	Frequency (*N*)	Cumulated incidence (*N* per 10,000 inhabitants)
< 1 year	320	296
1–4 years	168	52
5–14 years	159	15
15–44 years	317	12
45–59 years	132	7
60–74 years	243	21
≥ 75 years	497	80
Total	1,836	23

### Clinical features and severity.

Mean and median hospital stay durations were 8 and 4 days, respectively. We reported an overall 1,821 hospitalized cases infected through vector-borne transmission, including 1,191 cases recorded in Martinique, 630 cases in Guadeloupe, and 15 from MTCT transmission.

Among all hospitalized cases, 361 (20.4% of total hospitalized cases) were severe, 354 of whom resulted from vector-borne transmission, and seven from MTCT.

Severity was assessed in 361 hospitalized patients accounting for 11.7 per 10,000 suspected CHIKV cases. One hundred and thirty-five cases (7.4% of total hospitalized cases) were admitted to an intensive care unit accounting for 37.4% of total severe cases (Table 2).

**Table 2:** Number of hospitalized cases by mode of transmission and by clinical severity during the chikungunya virus epidemics in Martinique and Guadeloupe—from December 2013 to January 2

Clinical features of chikungunya	*N*	Proportion (%)
Non-neonatal transmission	1,821	99.2
*Nonsevere*	*1,467*	*79.9*
*Typical features*	*793*	*43.2*
*Atypical features*	*674*	*36.7*
*Severe*	*354*	*19.3*
Neonatal transmission	15	0.8
*Nonsevere neonatal features*	*8*	*0.4*
*Severe neonatal features*	*7*	*0.4*
Total	1,836	100.0

At least one underlying health condition was present in 51% of total hospitalized cases, such as diabetes (16% of total cases and 29% of severe cases had diabetes), sickle cell anemia (4%), or immunosuppression (1.5%).

Among non-MTCT cases (*N* = 1,821), 90% had fever and 71% presented arthralgia. Other reported symptoms were myalgia (28%), rash (21%), headache (20%), nausea (19%), edema (18%), abdominal pain (12%), diarrhea (11%), itching (6%), and tenosynovitis (1%).

We reported specific syndromes affecting atypical and severe cases. The most frequent atypical syndrome was neurological disorder (40%) with seizure that affected 156 cases (8.6% of total), most of whom were children (78%), and encephalitis reported in 38 cases (3.7% of total), most of whom were adults (79%). Guillain–Barré syndrome was diagnosed in 13 cases (0.7% of total) of which seven were in Martinique and six in Guadeloupe.

Acute cardiovascular disorders (27%) and renal impairments (26%) were also reported. Other syndromes, such as respiratory disorder (22%), intense pain syndrome (21%), severe liver dysfunction (12%), hemorrhagic manifestations (10%), dermatologic manifestations, other than typical manifestations (10%), such as bullous dermatosis and severe digestive disorder (3%), were also notified in atypical or severe cases.

The clinical features notified in non-MTCT severe cases were cardiovascular system failures in almost half of the cases (46%) followed by renal (36%), respiratory (35%), and cerebral (31%) serious disorders. Finally, liver failure (14%) and other type of organ failures (5%) were less frequently reported.

### Chikungunya and pregnancy.

We reported 185 hospitalized pregnant women, 66 in Martinique and 119 in Guadeloupe. The main reasons for hospitalization were womb contractions and high-grade fever. Pregnant women accounted for 10.1% of all 1,836 hospitalized cases. Overall mean and median ages of hospitalized pregnant women were 29 years (range: 15–45).

Eighteen of the 185 hospitalized pregnant women (9.7%) developed atypical nonsevere manifestations and three (1.6%) presented with severe clinical features. Mean and median hospitalization stay duration were 6 and 3 days (range: 1–50 days).

### MTCT cases.

Fifteen MTCT cases were reported, five in Martinique and 10 in Guadeloupe. The mean age at symptoms onset was 6 days (median: 5 days), and 14 cases (93%) were observed in boys and one case in a girl (7%). The mean of hospitalization duration stay was 12 days and median was 9 days (range: 5–27 days). Seven MTCT cases developed serious clinical manifestations, four of which presented with cardiovascular failures. No death was reported in MTCT cases.

### Features of non-MTCT severe and nonsevere cases.

Among the 1,821 non-MTCT cases, proportions of cases aged 60 years or older were greater in severe cases (69.5%) compared with nonsevere cases (33.6%) (*P* < 0.001). Indeed, mean and median ages in severe cases were 63 and 72 years, whereas they were 37 and 29 years in nonsevere cases, respectively. Among severe cases, 77% had at least one comorbidity, whereas only 46% in nonsevere cases (*P* < 0.001). As expected, the duration of hospitalization increased significantly with severity: mean of hospitalization stay duration in severe cases was 14 days, (median: 10 days) whereas the mean in nonsevere cases was 6 days (median: 4 days) (*P* < 0.001) (Table 3).

**Table 3:** Characteristics of chikungunya virus hospitalized cases by severity in patients aged more than 10 days—Martinique and Guadeloupe— from December 2013 to January 2015

	Nonsevere cases		Severe cases	
Age groups	*N*	(%)	*N*	(%)
< 1 year	278	19	27	7.6
1–4 years	160	10.9	8	2.3
5–14 years	148	10.1	11	3.1
15–44 years	288	19.6	29	8.2
45–59 years	99	6.7	33	9.3
60–74 years	156	10.6	87	24.6
≥ 75 years	338	23.0	159	44.9
Mean age (years)	36.6		62.8	
Sex				
Females	808	55.1	157	44.5
Hospitalization duration				
≤ 72 hours	711	48.5	70	19.9
Underlying comorbidity				
≥ 1 comorbidity				
Yes	670	45.7	274	77.4
Diabetes				
Yes	196	13.4	102	28.8
Sickle cell anemia				
Yes	62	4.3	11	3.1
Immunosuppression				
Yes	21	1.4	7	2.0
Other comorbidities				
Yes	570	38.9	257	72.6

* NS cases = nonsevere cases; S cases = severe cases.

### Mortality.

During this epidemic, we reported a total of 74 deaths among hospitalized cases. The mean age of deceased persons was 75 years (median: 81 years; range: 0–101 years). However, seven deaths were recorded in patients aged less than 60 years of age, including two infants (< 1 year old). The sex ratio was 1.5, and 63 deceased patients among the 74 reported deaths (85%) had at least one comorbidity, such as diabetes in almost 30% of the deceased patients. Causes of deaths were mostly cardiovascular failures reported in 57 patients (77%). Respiratory, renal, cerebral, and hepatic failures occurred respectively in 58.1%, 55.4%, 50%, 24.3%, and other organ failures in 4% of patients. The mean and median of hospitalization duration stay were 9 and 7 days (range: 0–46 days). Four percent of the total hospitalized CHIKV cases died and the fatality reported for the estimated CHIKV infected population was 4.8 per 10,000 suspected CHIKV clinical cases.

## DISCUSSION

The emergence of chikungunya in the Caribbean in December 2013 was the start of the first reported epidemic caused by CHIKV in this part of the world. We set up an active hospital-based surveillance system in two French Caribbean territories to assess severity of the phenomenon and to detect atypical forms of the disease. Our main results show that a total of 1,836 CHIKV cases were hospitalized in Martinique and Guadeloupe: the estimated hospitalization rate was 60 per 10.000 CHIKV clinical cases. Severity rate accounted for approximately 12 per 10,000 CHIKV clinical cases and fatality rate for approximately 5 per 10,000 CHIKV clinical cases. Our results also emphasized MTCT occurrence during the epidemic with half of the MTCT cases being severe.

We targeted all major, acute, or emergency wards of public hospitals as severe cases would be mostly cared for in public hospitals. Official hospital data emphasize this hypothesis as public and private hospital activities indicate that 78% of the total number of hospital stays in medical departments rely on public hospitals (95% in Martinique and 62% in Guadeloupe). Therefore, it is unlikely that severity estimates based on the assumption that severe cases would mainly be treated in public hospitals have been biased by the setting of this observational study.

Severe clinical forms of the infection arose in Reunion Island during the massive CHIKV epidemic.^15,18^ Although Reunion Island and Martinique/Guadeloupe have similar demographic sizes with, respectively, 785,000 and 785,076 inhabitants and similar medical infrastructures, the hospital surveillance system set up in the Caribbean captured more cases. In comparing the estimated population at risk for hospitalization in both regions during the epidemics, this population was larger in Martinique and Guadeloupe as an estimated additional 70,000 suspected CHIKV cases arose compared with that of Reunion Island. Indeed, in La Reunion, a total of 878 hospitalized cases were reported out of an estimated 244,000 suspected CHIKV cases (36 per 10,000 CHIKV cases) occurring in the background population whereas in the two French Caribbean territories, 1,836 cases were admitted to hospital out of an estimated 308,000 suspected CHIKV cases (60 per 10,000 CHIKV cases).^19^ Furthermore, sensitivity of the French Caribbean hospital surveillance was greater than that in Reunion island as any symptomatic biologically confirmed case was included in the first system, whereas patients with symptoms limited to fever and arthralgia were excluded from the second system.^15^ Other factors might have played a role in the difference, such as diagnostic and hospitalization practices differing across countries.

Severe clinical forms of the infection arose in Reunion Island during the massive CHIKV epidemic. We also reported severe cases with fatal issues as reported in the Caribbean and Latin American countries affected by CHIKV epidemics during the same period.^20,21^ Although CHIKV infection has commonly been considered a mild disease, the growing number of publications and reports from different countries underlines the potential severity of the infection. We emphasized that the incidence rate of severe cases in Reunion Island was similar to that in Martinique and Guadeloupe despite the fact that the genotypes circulating in the two regions (Indian Ocean and the Caribbean) were different. Incidence and fatality rate of severe hospitalized cases were respectively 10 and 2.9 per 10,000 suspected CHIKV cases in Reunion Island, compared with 11.5 and 2.4 per 10,000 suspected CHIKV cases in pooled cases from Martinique and Guadeloupe. Lahariya and Pradhan^22^ notified in their review on the reemergence of CHIKV in India that the infection is severe in infants, in elderly, and in immunocompromised patients. We have not identified a greater severity in the very young especially in children contaminated through vector transmission although two deaths were reported. Economopoulou and Dominguez emphasized that among other risk factors, older age (> 85 years) was associated with increased mortality.^15,18^ Our results also suggested that deaths occurred mostly in elderly persons although five deaths were recorded in patients aged more than 1 year and less than 60 years of age. Comorbidity might be associated with severity as we reported higher proportion of comorbidity in severe cases compared with others. Investigations of risk factors in Reunion Island highlighted that cardio-vascular, respiratory disorders or hypertension were associated with increased risk of severe illness.^15,18^ Furthermore, in our study diabetes was reported in 29% of severe clinical cases whereas prevalence of diabetes was estimated to be 5.6% and 6.9% in Martinique and Guadeloupe, respectively.^23^ The observed discrepancy between diabetes prevalence in the general population and burden of diabetes in our study population suggests that diabetes could play a role in the clinical course of the infection or in hospitalization practices. The same finding was underlined in a previous publication on chikungunya.^24^ Further investigations are needed to explore whether diabetes is a risk factor of severity especially in countries where diabetes is a major public health concern, such as in the Caribbean.

Regarding clinical signs, fever and arthralgia were the most frequently reported symptoms as widely described in the literature.^2,24–28^ The atypical or severe clinical features affecting our patients were mainly neurological, cardiovascular, or renal disorders. Among cases with neurological manifestations, Guillain–Barré syndrome cases, seizure in children, and encephalitis in adults were reported. We hypothesized that Guillain–Barré syndrome might have occurred more frequently or have simply been more frequently reported compared with previous epidemics.^18,29^ Whether the neurovirulence of the circulating Asian genotype led to greater neurological impact in comparison to that circulating earlier might be hypothesized. However, further clinical and epidemiological investigations are needed to explore this hypothesis. Although the disease was benign in most CHIKV cases occurring in the two French Caribbean islands of Martinique and Guadeloupe, severe and fatal outcomes were reported. In adults, occurrence of death was frequently related to the decompensation of an underlying disease and mostly indirectly caused by the CHIKV infection.

Another interesting finding is that 80% of hospitalized cases were nonsevere cases. This result suggests that given the context of an emerging infection, the debilitating nature of the disease, and uncertainty on clinical evolution including occurrence of deaths, clinicians unfamiliar with the infection were probably more likely to hospitalize CHIKV infected patients including those without severe clinical presentations.^26,30^

Fifteen neonatal cases consecutive to MTCT occurred in Martinique and Guadeloupe, confirming vertical CHIKV transmission.^31,32^ Almost half of reported MTCT cases in Martinique and Guadeloupe were severe cases. This result is concordant with study results on vertical transmission in Colombia, Salvador, and Dominican Republic emphasizing that severity also occurred in neonates born from CHIKV infected mothers. In Reunion Island where vertical transmission was first reported, a higher number of MTCT cases were confirmed (44 MTCT cases) despite the smaller magnitude of the epidemic.^18^ With enhanced health communication on the risk of vertical transmission already known during the 2013–2015 Caribbean epidemics, smaller birth cohorts in the French Caribbean (9,200 births in 2013) compared with those of Reunion Island (14,800 births in 2005) might explain the lower recorded neonatal cases consecutive to MTCT. Regarding the high sex ratio of MTCT cases with boys being overrepresented, we cannot provide any relevant explanation as gender difference practices in newborns are very uncommon in this setting.

Awareness on possible severity and deaths associated with the infection has grown over the past decade as chikungunya has been reemerging in different parts of the world where the competent vectors are endemic. Adequate measures and prevention could alleviate the health impact of this viral infection in population groups most at risk: infants, the elderly, persons with underlying health conditions, and pregnant women. Enhanced vector control strategies and vaccine research initiated with promising preliminary results should be strongly supported to empower public health stakeholders with a wider and more effective range of preventive tools.^33^
